# Tibial nerve stimulation for low anterior resection syndrome after rectal cancer surgery: current evidence, patient-tailored clinical positioning, and future directions

**DOI:** 10.3389/fsurg.2026.1945654

**Published:** 2026-10-06

**Authors:** Tao He, Jia Hu, Fangqiong Xu, Jian Huang

**Affiliations:** Department of Surgical Oncology, The First Affiliated Hospital of Kunming Medical University, Kunming, Yunnan, China

**Keywords:** fecal incontinence, low anterior resection syndrome, percutaneous tibial nerve stimulation, rectal cancer, transcutaneous tibial nerve stimulation

## Abstract

Low anterior resection syndrome (LARS) is a common long-term bowel dysfunction following rectal cancer sphincter-preserving surgery, mainly manifested as defecation urgency, frequency, clustered defecation, fecal incontinence, and a feeling of incomplete evacuation, significantly impacting patients' quality of life. Current treatments largely follow symptom-oriented and stepwise management principles, including dietary and behavioral adjustments, medication, pelvic floor rehabilitation, biofeedback, and transanal irrigation. However, efficacy remains unsatisfactory for patients with persistent major LARS or refractory symptoms. Tibial nerve stimulation (TNS), as a minimally invasive or non-invasive neuromodulation method, has gradually gained attention in recent years. Direct LARS-specific studies of percutaneous tibial nerve stimulation (PTNS) suggest a potential therapeutic signal. However, the small sample sizes, inconsistent stimulation protocols, inconsistent comparative findings, and limited follow-up preclude considering PTNS an established escalation treatment. Transcutaneous tibial nerve stimulation (TTNS) offers advantages such as being non-invasive, repeatable, and potentially suitable for home maintenance therapy. However, direct evidence for its use in LARS remains limited, and it cannot currently be recommended as equivalent to PTNS. This article reviews the clinical characteristics of LARS, the basis of TNS's role, research evidence for PTNS and TTNS, their clinical positioning, and future research directions, aiming to provide a reference for individualized treatment and subsequent clinical research of LARS.

## 1 Introduction

Colorectal cancer has a heavy burden in China. In 2022, the total number of colorectal cancer cases reached 517,100, ranking second among malignant tumors in China (1). With the development of total mesorectal excision, neoadjuvant therapy and low anastomosis, more rectal cancer patients have been able to have the opportunity to preserve their anus. However, preserving the anus does not mean preserving normal defecation function. Low anterior resection syndrome (LARS) can occur in a considerable number of rectal cancer patients after anastomosis, becoming an important issue affecting the long-term quality of life after surgery. It is common in patients with low anastomosis, those receiving radiotherapy or those undergoing temporary protective stomas (2, 3). Postoperative bowel dysfunction is particularly important after very low sphincter-preserving surgery. A 2026 systematic review and meta-analysis of 18 studies involving 1,337 patients after intersphincteric resection reported a pooled prevalence of major LARS of 46% (95% CI, 33%–60%) (4). In a contemporary two-center cohort, major LARS occurred in 61 of 128 patients (47.7%) assessed 12–15 months after ileostomy reversal following hand-sewn intersphincteric resection (5). At present, the treatment strategy for LARS is symptom-oriented, following the principles of stepwise and individualized treatment, but there is a lack of a single best intervention that is applicable to all patients. Among them, sacral neuromodulation (SNM) is usually used as an upgraded treatment option for patients with major LARS or refractory symptoms, but its invasiveness, high cost, and reliance on stimulus response assessment limit its clinical promotion. Tibial nerve stimulation (TNS) has gained attention in recent years due to its less invasive nature compared to SNM and its potential for maintenance therapy in outpatient or home settings. This article reviews the current evidence on TNS in the management of low anterior resection syndrome (LARS), focusing on LARS-specific clinical studies, systematic reviews, meta-analyses, and management recommendations. Studies on fecal incontinence and overactive bladder are also used as indirect evidence to discuss the neuromodulation basis, feasibility, and extrapolation boundaries of TNS.

## 2 Literature search and evidence selection

This narrative review was based on a targeted literature search of PubMed/MEDLINE, Web of Science Core Collection, and the Cochrane Library from database inception through August 2026. Search terms combined “low anterior resection syndrome,” “LARS,” or “anterior resection syndrome” with “tibial nerve stimulation,” “posterior tibial nerve stimulation,” “percutaneous tibial nerve stimulation,” “transcutaneous tibial nerve stimulation,” “PTNS,” or “TTNS.” Reference lists of relevant studies and reviews were also screened, and guidelines and consensus statements were identified through targeted searches of professional-society and journal websites.

Evidence was organized into three categories according to its directness to LARS: (1) direct LARS-specific clinical studies of PTNS or TTNS; (2) LARS-focused reviews, systematic reviews, meta-analyses, guidelines, and consensus statements; and (3) indirect evidence from fecal incontinence and overactive bladder. Within the direct evidence, randomized or comparative studies were given greater interpretive weight than uncontrolled observational studies. Indirect evidence was used only to discuss biological plausibility, stimulation methods, feasibility, and safety, and not to infer clinical effectiveness in LARS. No formal risk-of-bias assessment or quantitative evidence synthesis was undertaken. The main characteristics, outcomes, and limitations of direct LARS-specific clinical studies of PTNS and TTNS are summarized in Table 1.

**Table 1:** Direct LARS-specific clinical studies of PTNS and TTNS.

Study	Design	Sample size	LARS severity	Stimulation protocol	Comparator	Follow-up	Main outcomes	Major limitations
Vigorita et al., 2017 (22)	Prospective single-arm pilot	*n* = 10 (7 completed)	LARS score ≥21	PTNS, 30 min, twice weekly ×6 weeks; responder extension	None	Short-term; responder-based	LARS improved in 5/10; 2/10 had no LARS after treatment	Small, uncontrolled; 3 non-completers; durability uncertain
Altomare et al., 2017 (23)	Prospective two-centre single-arm pilot	*n* = 21	Median LARS score 32	PTNS, 12 × 30 min: twice weekly ×4 weeks, then weekly ×4 weeks	None	6 months	LARS: 32 → 27 (*P* = 0.009); >50% St Mark's improvement: 9/21; TAPE: 55 → 58 (*P* = 0.004)	Small, uncontrolled; multiple outcomes
Enríquez-Navascués et al., 2020 (24)	Single-centre open-label RCT	Randomized: TAI 13, PTNS 14; completed: 10 and 13	Chronic major LARS (>29)	PTNS, 20 × 30 min: 12 weekly, 4 fortnightly, 4 monthly	TAI	24 weeks	LARS-category improvement—ITT: 8/13 vs. 4/14; PP: 8/10 vs. 3/13. > 50% Vaizey reduction: 8/10 vs. 5/13 (TAI vs. PTNS)	Small, open-label exploratory trial; attrition
Cuicchi et al., 2020 (25)	Single-centre randomized pilot	*n* = 12 (6/arm); 10 at 1 year	LARS score ≥21	PTNS, 17 × 30 min including top-up sessions	Medical therapy	1 year	End-treatment PTNS arm: LARS 35.8 → 28.2; FISI 36.8 → 17.2 (both *P* = 0.02); maintained at 1 year in available cases	*n* = 6/arm; unblinded; underpowered; incomplete 1-year data
Marinello et al., 2021 (26)	Multicentre double-blind sham RCT	*n* = 46 (23/arm)	Major LARS	PTNS, 16 × 30 min: 12 weekly + 4 fortnightly	Sham stimulation	12 months	PTNS within-group at 12 months: LARS 36.4 → 30.7; St Mark's 15.4 → 12.5 (both *P* = 0.018); no between-group LARS difference	Small; differential attrition; no superiority vs. sham; phenotype not prespecified
Zhang et al., 2024 (33)	Retrospective uncontrolled pre–post study	*n* = 65; 62 completed	42 minor LARS; 20 major LARS	Bilateral TTNS, 15 Hz, 0.1–1.0 mA, 30 min, 3×/week × 3 months	None	3 months	LARS 36.4 → 26.1; Wexner 8.3 → 6.8 (both *P* < 0.001); no serious adverse events	Retrospective, uncontrolled; short follow-up; no causal or PTNS-equivalence inference

FISI, fecal incontinence severity index; ITT, intention-to-treat; LARS, low anterior resection syndrome; PP, per-protocol; PTNS, percutaneous tibial nerve stimulation; RCT, randomized controlled trial; TAI, transanal irrigation; TAPE, three axial perineal evaluation; TTNS, transcutaneous tibial nerve stimulation. Arrows indicate changes from baseline to follow-up. Unless otherwise specified, *P* values refer to within-group comparisons.

## 3 Clinical characteristics, assessment, and treatment challenges of LARS

LARS is defined as a group of bowel dysfunction syndromes that occur after low anterior resection of rectal cancer (6). Its clinical phenotypes are significantly heterogeneous: some patients mainly present with urgency, frequency, clustered defecation and fecal incontinence; some patients mainly present with incomplete defecation, defecation obstruction or constipation-like symptoms; and some patients may alternate between the two symptom patterns. As the treatment of rectal cancer has shifted from pursuing tumor control to long-term quality of life management, LARS has been considered one of the most important long-term survival issues after rectal cancer surgery (3).

The LARS score, established by Emmertsen and Laurberg, is currently the most widely used symptom scale in clinical research and practice (7). After international validation, it has become an important tool for cross-study comparisons (8). However, relying solely on the LARS score cannot fully reflect the impact of LARS on patients' quality of life, psychological burden, social restrictions, and defecation dysfunction. Clinical management reviews suggest that symptom assessment tools and quality of life assessment tools should be used in combination in clinical practice and research (9). Objective detection methods such as anorectal manometry, defecography, anal canal ultrasound, and pelvic floor muscle function assessment may have application value in specific research or specialty scenarios. Therefore, TNS-related studies should not only rely on short-term changes in the LARS score, but should also include outcome indicators such as the Wexner score or St Mark incontinence score, Memorial Sloan Kettering Cancer Center bowel function scale (MSKCC-BFI), EORTC QLQ-C30/CR29, defecation diary, patient satisfaction, treatment burden, compliance, and adverse events.

At present, the treatment principle of LARS is mainly based on symptomatic support, individualized management and step-by-step intervention. The MANUEL project proposed a management framework for LARS, which starts with dietary adjustment, behavioral intervention and drug treatment, and gradually upgrades to pelvic floor rehabilitation, biofeedback, transanal irrigation (TAI) and neuromodulation according to the severity of symptoms and the needs of patients (10). Two authoritative consensus documents released in China in 2025—the Chinese Expert Consensus on the Diagnosis and Treatment of Low Anterior Resection Syndrome (2025 Edition) (11) and the Expert Consensus on the Diagnosis and Treatment of Bowel Dysfunction after Low Anterior Resection (2025 Edition) (12)—also recommend individualized and step-by-step management based on symptom phenotype (stool storage disorder, emptying disorder, mixed disorder), and include neuromodulation in the upgraded treatment options for refractory LARS. The BOREAL pilot study also emphasizes that it is of great significance to carry out structured bowel rehabilitation after total mesorectal excision, the core of which is early identification, continuous assessment and sequential intervention (13). Previous reviews have summarized various treatment methods for LARS, including pelvic floor rehabilitation, TAI, sacral nerve stimulation, PTNS, probiotics and 5-HT3 receptor antagonists, but have also consistently pointed out that the overall quality of existing evidence is low and there is a lack of large-sample, multicenter randomized controlled studies (14). A recent umbrella review suggests that TAI and pelvic floor rehabilitation can be used as basic treatment methods, while SNM or PTNS can be considered for refractory cases, especially patients with fecal incontinence as the main manifestation (15).

However, the main challenge in treating LARS lies in the significant heterogeneity of symptom phenotypes and pathophysiological mechanisms. Some patients primarily experience urgency, fecal incontinence, and decreased bowel control, while others primarily experience incomplete defecation, difficulty emptying the bowels, or constipation-like symptoms. Different symptom types may respond differently to diet, medication, pelvic floor rehabilitation, TAI, or neuromodulation. Therefore, while the existing stepwise treatment framework provides a pathway for clinical management, there is no single optimal intervention applicable to all LARS patients, and patients with major LARS or treatment-resistant symptoms still experience substantial impairment and impaired quality of life even after receiving standard treatment.

Against this backdrop, the potential value of TNS has gradually attracted attention, but its specific position in the LARS stepwise treatment still needs further clarification. Compared with SNM, TNS is less invasive, relatively easy to operate, and has the potential for outpatient treatment or home maintenance; however, compared with basic treatments such as dietary adjustment, drug therapy, pelvic floor rehabilitation and TAI, its evidence base is still insufficient, and its efficacy may be affected by factors such as dominant symptom phenotype, degree of nerve damage, treatment parameters and follow-up time. Therefore, TNS may be considered a promising or selective adjunct for carefully selected patients with persistent symptoms after conventional treatment (16).

## 4 Theoretical basis and implementation method of tibial nerve stimulation

Sacral neuromodulation (SNM) can improve symptoms in some LARS patients. Its mechanism may be through the modulation of sacral reflex pathways, pelvic floor synergistic movement, anorectal sensory and defecation control- related neural pathways. However, SNM requires surgical implantation, is expensive, and depends on strict indication selection and trial stimulation evaluation (17, 18). In contrast, TNS is less invasive and relatively simple to operate, thus becoming an important direction for exploration in the neuromodulation treatment of LARS. The tibial nerve mainly originates from the L4-S3 nerve root and overlaps with the neural pathways related to bladder, pelvic floor, anal sphincter and rectal sensorimotor function, providing a theoretical basis for nerve stimulation therapy for defecation control disorders (19). Early research on electrical stimulation therapy for detrusor instability also promoted the application of tibial nerve modulation in urological and pelvic floor diseases (20). Although the pathogenesis of LARS differs from that of overactive bladder and non-tumor-related fecal incontinence, the common sacral reflex mechanism provides a reasonable basis for the application of TNS in the treatment of LARS. However, mechanistic overlap supports the biological plausibility of studying TNS in LARS, it does not establish clinical effectiveness in the LARS population.

PTNS is not “implantable tibial nerve stimulator treatment”. Instead, a needle electrode is inserted above the medial malleolus along the course of the posterior tibial nerve and connected to an external stimulator. The common course of treatment is once a week, 30 min each time, for 6 to 12 weeks. TTNS, on the other hand, uses a surface electrode attached to the corresponding area to provide stimulation. It does not require puncture and is more convenient. The treatment parameters are mostly based on PTNS. Theoretically, it is suitable for outpatient extension or home maintenance treatment (16, 21).

## 5 PTNS original research evidence

Among the various tibial nerve stimulation methods currently available, PTNS is the treatment method with relatively more direct evidence in the field of LARS treatment. The exploratory study conducted by Vigorita et al. first applied PTNS to LARS patients, suggesting that the therapy has certain feasibility and may improve some bowel control-related symptoms and quality of life, but it was a small sample single-arm design and could not draw definitive conclusions (22). The prospective pilot study by Altomare et al. enrolled 21 patients who underwent 12 PTNS sessions of 30 min each. At 6 months, the median LARS Score decreased from 32 to 27 (*P* = 0.009), and the median Three Axial Perineal Evaluation (TAPE) score increased from 55 to 58 (*P* = 0.004). The authors proposed the TAPE score as a complementary assessment when urinary or sexual dysfunction coexists (23). The above early studies confirmed that the therapy has potential benefit signals, but the exploratory design and small sample size limited the strength of its evidence.

Subsequent comparative studies were carried out: Enriquez- Navascues et al. conducted a randomized controlled trial comparing TAI and PTNS in the treatment of chronic major LARS (TAI, *n* = 13; PTNS, *n* = 14). Four patients were excluded because of intercurrent disease or early dropout, leaving 23 patients for analysis (TAI, *n* = 10; PTNS, *n* = 13). The results showed that both interventions could improve symptoms, but TAI had a more significant reduction rate and improvement range. In the TAI group, 8 out of 10 patients (80%) experienced symptom reduction and their Vaizey incontinence scores decreased from 15 (IQR, 11–18) to 6 (IQR, 4–7) (*P* = 0.037), while in the PTNS group, only 5 out of 13 patients (38%) experienced symptom reduction and their Vaizey scores decreased from 14 to 9 (*P* = 0.007). In both groups, 80% and 38% of patients, respectively, experienced a reduction in incontinence scores of more than 50% (24). This result suggests that although PTNS has a therapeutic signal, the improvement range may not be as good as that of TAI, and the applicable population for different interventions still needs further clarification. Cuicchi et al. conducted a randomized preliminary trial (6 patients in the PTNS combined with drug treatment group and 6 patients in the drug treatment group alone) and followed up for 1 year. The LARS score and incontinence score (FISI scale decreased from 36.8 to 17.2) in the PTNS group showed an improvement trend, suggesting that PTNS may bring about symptom improvement. However, the sample size was too small and the long-term efficacy was still unclear (25). Marinello et al. conducted a randomized clinical trial with sham stimulation control for patients with major LARS (23 patients in the PTNS group and 23 patients in the sham stimulation group). The primary endpoint was the LARS score at 12 months after treatment. Both groups showed an early decrease in LARS scores, but only the PTNS group maintained long-term benefit (LARS score decreased from 36.4 ± 3.9 at baseline to 30.7 ± 11.5 at 12 months, *P* = 0.018; effect size decreased by about 15.7%). In addition, the St Mark's incontinence score in the PTNS group decreased from 15.4 ± 5.2 at baseline to 12.5 ± 6.4 at 12 months (*P* = 0.018). Although this within-group improvement supports further investigation, the trial was neither designed nor powered to determine whether patients with an incontinence-predominant LARS phenotype respond preferentially to PTNS. Therefore, these findings provide only preliminary support for further evaluation of PTNS in this patient subgroup (26).

In conclusion, PTNS has potential value in the treatment of LARS, but not all patients will benefit. Whether dominant symptom phenotype modifies the response to PTNS has not been prospectively established and should be examined in adequately powered studies with prespecified subgroup analyses.

## 6 Comprehensive evidence for PTNS treatment of LARS

Tazhikova et al.'s systematic review included 5 studies and 116 patients with LARS-related fecal incontinence. They analyzed the efficacy of TNS in patients with LARS-related fecal incontinence after colorectal cancer surgery and believed that this treatment method has potential application value. However, they also pointed out that the existing studies have small sample sizes, low credibility of evidence, inconsistent outcome indicators, and short follow-up periods (27). Liapis et al. specifically analyzed the role of PTNS in LARS and confirmed that it can improve LARS scores and some intestinal function indicators. However, the number of included studies was limited, the heterogeneity and risk of bias were high, and the evidence strength of the conclusions was insufficient (28).

Some studies have offered more cautious explanations. A systematic review and meta-analysis of randomized controlled trials showed that PTNS did not consistently demonstrate a statistical advantage over all control protocols in terms of LARS scores and related outcomes of fecal incontinence (29). A network meta-analysis further suggested that the efficacy of different interventions varied at different follow-up times and for different outcome indicators: short-term (≤6 months) transanal irrigation (TAI) showed the most significant improvement in overall LARS symptoms (MD, −14.13, 95% CrI, −20.11 to −7.83) and incontinence severity (SMD, −1.34, 95% CrI, −1.97 to −0.71); long-term (>6 months) TAI was still most likely to rank first, followed by PTNS, and then pelvic floor rehabilitation, but the differences between the groups were not statistically significant (30, 31). A recent systematic review focusing on treatment options (including 38 articles and 1,914 patients) also pointed out that no single intervention can consistently outperform all LARS-related outcomes, and clinical treatment should be selected based on individualized symptoms (32).

In summary, PTNS has biological rationale and a potential therapeutic signal in LARS, but efficacy has not been consistent across the available evidence. Accordingly, PTNS should be presented as a promising or selective option rather than an established escalation treatment.

## 7 Direct LARS-specific evidence for TTNS

Zhang Jianhua et al. reported an early clinical observation of 62 patients with LARS after rectal cancer surgery treated with TTNS. The results showed that the LARS score, Wexner score, bowel function and some quality of life indicators improved after treatment compared with the baseline, and no serious adverse events occurred (33). This uncontrolled study suggests that TTNS is feasible and was associated with preliminary pre–post improvements in LARS-related outcomes. However, it is a single-center, single-arm observational study, lacking sham stimulation control trials, positive control trials and long-term follow-up data, and the level of evidence is still limited.

## 8 Indirect evidence from fecal incontinence and overactive bladder

Because direct LARS-specific evidence for TTNS is limited, studies in other pelvic-floor disorders can inform biological plausibility, feasibility, and safety, but cannot establish effectiveness in LARS. Studies using TNS to treat female idiopathic overactive bladder have shown that both PTNS and TTNS stimulation methods are superior to simple bladder training and can improve symptoms and quality of life, with similar overall efficacy (21, 34, 35). A review conducted by Tah masbi et al. confirmed that tibial nerve stimulation may improve some defecation habits and quality of life outcomes in patients with fecal incontinence, but the results were heterogeneous, with small sample size, mixed etiologies, and insufficient follow-up time (36).

It is worth noting that the evidence for TTNS in the field of fecal incontinence is not entirely positive. The multicenter, double-blind, randomized sham-stimulation controlled trial conducted by Leroi et al. (including 144 patients with fecal incontinence) failed to demonstrate that TTNS has a stable advantage over sham stimulation (37). George et al. directly compared PTNS, TTNS and sham stimulation in the treatment of fecal incontinence, and the results showed that some TTNS subjects experienced a reduction of at least 50% in the number of fecal incontinence episodes, but the overall response seemed to be weaker than PTNS (38). Therefore, the evidence for fecal incontinence can support the theoretical feasibility of TTNS for the treatment of fecal incontinence disorders. However, the pathogenesis of LARS involves multiple factors such as decreased neorectal pouch capacity, low anastomosis, radiation damage, pelvic autonomic nerve damage, anal sphincter dysfunction and abnormal intestinal motility, and cannot be simply equated with general fecal incontinence. This indirect evidence can provide a basis for TTNS to conduct LARS-specific research, but it cannot replace the evidence of LARS-specific randomized controlled trials.

## 9 Clinical positioning of TNS

Based on existing evidence, TNS may instead be considered a promising or selective adjunct after conventional options have been assessed. For patients with minor LARS, dietary adjustments, bowel habit training, drug therapy, and lifestyle interventions should still be the initial management measures (10, 15, 16, 32), and this stepwise approach is consistent with the recommendations of the two LARS expert consensus documents in China by 2025 (11, 12). For patients with major LARS who have persistent symptoms, pelvic floor rehabilitation, biofeedback, and transanal irrigation can be further considered. PTNS may be considered as a selective adjunct for persistent symptoms after conventional treatment; however, preferential benefit in urgency- or incontinence-dominant LARS has not been prospectively established. Compared to PTNS, TTNS has the potential for non-invasive and home-based treatment, but its specific evidence in the treatment of LARS is still insufficient. Direct LARS-specific evidence for TTNS remains very limited. TTNS may represent a promising non-invasive approach that warrants further investigation, but it should not currently be considered an established alternative to PTNS. The 2025 Chinese consensus documents include percutaneous tibial nerve stimulation (PTNS) as a non-surgical neuromodulation option within stepwise LARS management, although the supporting evidence remains limited (11, 12). This aligns with the stepwise positioning of TNS in this paper. Future research should be based on symptom phenotypic stratification, and should not analyze all LARS patients as a homogeneous population, in order to clarify which patients may benefit from TNS.

Compared to SNM, PTNS and TTNS are generally less invasive, but appropriate patient selection and safety monitoring are still necessary. PTNS may cause puncture site pain, ecchymosis, bleeding, local paresthesia, or, rarely, infection. TTNS may cause skin irritation, discomfort, inaccurate electrode placement, or poor long-term adherence (22–26, 33, 36–38). Careful pre-treatment evaluation is necessary for patients receiving anticoagulation therapy, with local skin lesions, severe peripheral neuropathy, implanted pacemakers, or who are pregnant.

Adherence and treatment burden are core issues in the implementation of TNS. PTNS typically requires multiple outpatient visits and may be limited by transportation, cost, time, and medical resources. TTNS has the potential for home treatment, but its efficacy depends on the patient's ability to correctly place electrodes, achieve appropriate stimulation intensity, adhere to the prescribed frequency and duration, and maintain long-term adherence. If TTNS is to be used for long-term maintenance therapy in the future, training and adherence monitoring will be necessary.

## 10 Existing problems and future research directions

Current research on TNS treatment for LARS still faces several common challenges. First, most studies have small sample sizes, and inclusion criteria, baseline severity, symptom phenotypes, and follow-up time points are not entirely consistent, resulting in limited comparability between different studies (27–32). Second, stimulation parameters, treatment duration, and maintenance therapy protocols have not been standardized, making it difficult to reach a unified conclusion on efficacy evaluation (16, 28, 29). Third, existing studies mostly evaluate LARS as a whole syndrome, lacking sufficient analysis of differentiated responses to different symptom phenotypes—such as predominantly fecal incontinence, predominantly fecal aggregation, or predominantly fecal emptying disorder (16, 29). Based on current evidence, Whether tibial nerve stimulation provides greater benefit in incontinence- or urgency-dominant LARS remains a hypothesis that requires prospective confirmation in phenotype-stratified studies (26, 31, 32). Furthermore, data on the long-term efficacy, safety, compliance, cost-effectiveness, and head-to-head comparisons of TNS with treatments such as TAI, pelvic floor rehabilitation, and SNM remain insufficient (17, 18, 29–32).

Future research can be advanced in the following aspects: First, conduct more comprehensive, multi-center, large-sample, and blinded randomized controlled trials; second, standardize stimulation parameters, treatment duration, and outcome indicators, especially by incorporating LARS scores, fecal incontinence scores, quality of life scales, and patient satisfaction into the core outcome set; third, strengthen precision intervention research based on symptom phenotypes and risk stratification; and fourth, evaluate the feasibility and cost advantages of TTNS in home-based, long-term maintenance therapy. Only after these key questions are answered can the role of TNS in the tiered treatment of LARS become clearer.

## 11 Conclusion

LARS is a common and complex long-term bowel dysfunction after sphincter-preserving rectal cancer surgery, and management should remain symptom-oriented, individualized, and stepwise. Current LARS-specific evidence indicates a potential therapeutic signal for PTNS, but the small number of heterogeneous studies and inconsistent comparative findings preclude its characterization as an established escalation treatment. Its comparative effectiveness, optimal stimulation protocol, durability, and appropriate patient selection remain uncertain. Direct LARS-specific evidence for TTNS is largely limited to uncontrolled observation and is insufficient to establish clinical effectiveness or equivalence with PTNS. Multicenter trials with sham or active comparators, standardized outcomes, prespecified phenotype-stratified analyses, and long-term follow-up are needed.

## References

[1] Han B Zheng R Zeng H Wang S Sun K Chen R. Cancer incidence and mortality in China, 2022. J Natl Cancer Cent. (2024) 4:47–53. 10.1016/j.jncc.2024.01.006 39036382 PMC11256708

[2] Sun R Dai Z Zhang Y Lu J Zhang Y Xiao Y. The incidence and risk factors of low anterior resection syndrome (LARS) after sphincter-preserving surgery of rectal cancer: a systematic review and meta-analysis. Support Care Cancer. (2021) 29:7249–58. 10.1007/s00520-021-06326-2 34296335

[3] Ryoo S-B. Low anterior resection syndrome. Ann Gastroenterol Surg. (2023) 7:719–24. 10.1002/ags3.12695 37663958 PMC10472409

[4] Shamohammadi M Ullah SE Yiasemidou M Bahrdoust M Garavand AA Yarahmadi D. Low anterior resection syndrome after intersphincteric resection for low and ultralow rectal cancer: a systematic review and meta-analysis with narrative synthesis of reported factors. World J Surg Onc. (2026). [Advance online publication]. 10.1186/s12957-026-04526-w

[5] Shamohammadi M Noghlebari ZZ Alemrajabi M Madankan A Garavand AA Yarahmadi D. Major low anterior resection syndrome and fecal incontinence after ileostomy reversal following hand-sewn intersphincteric resection for low and ultralow rectal cancer. Surg Oncol. (2026) 68:102534. 10.1016/j.suronc.2026.102534 42604618

[6] Bryant CLC Lunniss PJ Knowles CH Thaha MA Chan CLH. Anterior resection syndrome. Lancet Oncol. (2012) 13:e403–408. 10.1016/S1470-2045(12)70236-X 22935240

[7] Emmertsen KJ Laurberg S. Low anterior resection syndrome score: development and validation of a symptom-based scoring system for bowel dysfunction after low anterior resection for rectal cancer. Ann Surg. (2012) 255:922–8. 10.1097/SLA.0b013e31824f1c21 22504191

[8] Juul T Ahlberg M Biondo S Emmertsen KJ Espin E Jimenez LM. International validation of the low anterior resection syndrome score. Ann Surg. (2014) 259:728–34. 10.1097/SLA.0b013e31828fac0b 23598379

[9] Zhang R Luo W Qiu Y Chen F Luo D Yang Y. Clinical management of low anterior resection syndrome: review of the current diagnosis and treatment. Cancers (Basel). (2023) 15:5011. 10.3390/cancers15205011 37894378 PMC10605930

[10] Christensen P IM Baeten C Espín-Basany E Martellucci J Nugent KP Zerbib F. Management guidelines for low anterior resection syndrome—the MANUEL project. Colorectal Dis. (2021) 23:461–75. 10.1111/codi.15517 33411977 PMC7986060

[11] Chinese Medical Doctor Association—Colorectal Surgeons Branch. Chinese expert consensus on the diagnosis and treatment of low anterior resection syndrome (2025 edition). Chin J Gen Surg. (2025) 34:1603–17. 10.7659/j.issn.1005-6947.250357

[12] Endoscopic Surgery Committee of Chinese Medical Doctor Association, Robotics and Laparoscopic Surgery Committee of Chinese Research Hospital Association, Editorial Board of Chinese Journal of Digestive Surgery. Expert consensus on the diagnosis and treatment of bowel dysfunction after low anterior resection (2025 edition). Chin J Dig Surg. (2025) 24:969–81. 10.3760/cma.j.cn115610-20250723-00485

[13] Harji D Fernandez B Boissieras L Berger A Capdepont M Zerbib F. A novel bowel rehabilitation programme after total mesorectal excision for rectal cancer: the BOREAL pilot study. Colorectal Dis. (2021) 23:2619–26. 10.1111/codi.15812 34264005

[14] Dulskas A Smolskas E Kildusiene I Samalavicius NE. Treatment possibilities for low anterior resection syndrome: a review of the literature. Int J Colorectal Dis. (2018) 33:251–60. 10.1007/s00384-017-2954-x 29313107

[15] Liu T Jiao X Hu C Su R Dong J Niu Q. Therapeutic strategies for low anterior resection syndrome: an umbrella review of systematic reviews. Int J Colorectal Dis. (2025) 40:171. 10.1007/s00384-025-04965-z 40762861 PMC12325548

[16] Rosen H Sebesta CG Sebesta C. Management of low anterior resection syndrome (LARS) following resection for rectal cancer. Cancers (Basel). (2023) 15:778. 10.3390/cancers15030778 36765736 PMC9913853

[17] Pires M Severo M Lopes A Neves S Matzel K Povo A. Sacral neuromodulation for low anterior resection syndrome: current status-a systematic review and meta-analysis. Int J Colorectal Dis. (2023) 38:189. 10.1007/s00384-023-04485-8 37428256

[18] Marinello F Fraccalvieri D Planellas P Adell Trapé M Gil JM Kreisler E. Sacral neuromodulation in patients with low anterior resection syndrome: the SANLARS randomized clinical trial. Dis Colon Rectum. (2024) 67:435–47. 10.1097/DCR.0000000000003143 38084933 PMC10846591

[19] Matzel KE Schmidt RA Tanagho EA. Neuroanatomy of the striated muscular anal continence mechanism. Implications for the use of neurostimulation. Dis Colon Rectum. (1990) 33:666–73. 10.1007/BF02150742 2376222

[20] Mcguire EJ Shi-Cun Z Horwinski ER Lytton B. Treatment of motor and sensory detrusor instability by electrical stimulation. J Urol. (1983) 129:78–9. 10.1016/s0022-5347(17)51928-x 6600794

[21] Sonmez R Yildiz N Alkan H. Efficacy of percutaneous and transcutaneous tibial nerve stimulation in women with idiopathic overactive bladder: a prospective randomised controlled trial. Ann Phys Rehabil Med. (2022) 65:101486. 10.1016/j.rehab.2021.101486 33429090

[22] Vigorita V Rausei S Troncoso Pereira P Trostchansky I Ruano Poblador A Moncada Iribarren E. A pilot study assessing the efficacy of posterior tibial nerve stimulation in the treatment of low anterior resection syndrome. Tech Coloproctol. (2017) 21:287–93. 10.1007/s10151-017-1608-x 28439675

[23] Altomare DF Picciariello A Ferrara C Digennaro R Ribas Y De Fazio M. Short-term outcome of percutaneous tibial nerve stimulation for low anterior resection syndrome: results of a pilot study. Colorectal Dis. (2017) 19:851–6. 10.1111/codi.13669 28371160

[24] Enriquez-Navascues JM Labaka-Arteaga I Aguirre-Allende I Artola-Etxeberria M Saralegui-Ansorena Y Elorza-Echaniz G. A randomized trial comparing transanal irrigation and percutaneous tibial nerve stimulation in the management of low anterior resection syndrome. Colorectal Dis. (2020) 22:303–9. 10.1111/codi.14870 31585495

[25] Cuicchi D Di Fabio F Guido A Llimpe FLR Morganti AG Ardizzoni A. Randomized pilot trial of percutaneous posterior tibial nerve stimulation versus medical therapy for the treatment of low anterior resection syndrome: one-year follow-up. Dis Colon Rectum. (2020) 63:1602–9. 10.1097/DCR.0000000000001614 33149022

[26] Marinello FG Jiménez LM Talavera E Fraccalvieri D Alberti P Ostiz F. Percutaneous tibial nerve stimulation in patients with severe low anterior resection syndrome: randomized clinical trial. Br J Surg. (2021) 108:380–7. 10.1093/bjs/znaa171 33793754

[27] Tazhikova A Makishev A Bekisheva A Dmitriyeva M Toleubayev M Sabitova A. Efficacy of tibial nerve stimulation on fecal incontinence in patients with low anterior resection syndrome following surgery for colorectal cancer. Ann Rehabil Med. (2022) 46:142–53. 10.5535/arm.22025 35793903 PMC9263329

[28] Liapis SC Baloyiannis I Perivoliotis K Lytras D Theodoropoulos G Tzovaras G. The role of percutaneous tibial nerve stimulation (PTNS) in low anterior resection syndrome (LARS): a systematic review and meta-analysis. J Gastrointest Cancer. (2023) 54:1128–39. 10.1007/s12029-023-00910-x 36703030

[29] Emile SH Garoufalia Z Barsom S Horesh N Gefen R Zhou P. Systematic review and meta-analysis of randomized clinical trials on the treatment of low anterior resection syndrome. Surgery. (2023) 173:1352–8. 10.1016/j.surg.2023.02.010 37012144

[30] Zhou L Zhang Z Wang L. Treatment of anterior resection syndrome: a systematic review and network meta-analysis. Eur J Surg Oncol. (2024) 50:108336. 10.1016/j.ejso.2024.108336 38626589

[31] Yu M Liu Y Li N Xu J Zhang H Li F. Optimal interventions for low anterior resection syndrome: Bayesian network meta-analysis of randomized controlled trials. Tech Coloproctol. (2025) 29:64. 10.1007/s10151-024-03099-8 39945891 PMC11825619

[32] Sharp G Findlay N Clark D Hong J. Systematic review of the management options available for low anterior resection syndrome (LARS). Tech Coloproctol. (2025) 29:58. 10.1007/s10151-024-03090-3 39903381

[33] Zhang J Zeng Y Wang X. Clinical observation on the efficacy of tibial nerve electrical stimulation with body surface electrodes in the treatment of 62 patients with low anterior resection syndrome of rectal cancer. Colorectal Anal Surg. (2024) 30:217–21. 10.19668/j.cnki.issn1674-0491.2024.02.016

[34] Ramírez-García I Blanco-Ratto L Kauffmann S Carralero-Martínez A Sánchez E. Efficacy of transcutaneous stimulation of the posterior tibial nerve compared to percutaneous stimulation in idiopathic overactive bladder syndrome: randomized control trial. Neurourol Urodyn. (2019) 38:261–8. 10.1002/nau.23843 30311692

[35] Ramírez-García I Kauffmann S Blanco-Ratto L Carralero-Martínez A Sánchez E. Patient-reported outcomes in the setting of a randomized control trial on the efficacy of transcutaneous stimulation of the posterior tibial nerve compared to percutaneous stimulation in idiopathic overactive bladder syndrome. Neurourol Urodyn. (2021) 40:295–302. 10.1002/nau.24554 33118624

[36] Tahmasbi F Mosaddeghi-Heris R Soleimanzadeh F Ghaderpanah R Sadrian SH Hajebrahimi S. Effects of posterior tibial nerve stimulation on fecal incontinence: an umbrella review. Neuromodulation. (2024) 27:229–42. 10.1016/j.neurom.2023.06.004 37452797

[37] Leroi AM Siproudhis L Etienney I Damon H Zerbib F Amarenco G. Transcutaneous electrical tibial nerve stimulation in the treatment of fecal incontinence: a randomized trial (CONSORT 1a). Am J Gastroenterol. (2012) 107:1888–96. 10.1038/ajg.2012.330 23032981

[38] George AT Kalmar K Sala S Kopanakis K Panarese A Dudding TC. Randomized controlled trial of percutaneous versus transcutaneous posterior tibial nerve stimulation in faecal incontinence. Br J Surg. (2013) 100:330–8. 10.1002/bjs.9000 23300071

